# Alginate lyase immobilized *Chlamydomonas* algae microrobots: minimally invasive therapy for biofilm penetration and eradication

**DOI:** 10.1016/j.apsb.2025.03.034

**Published:** 2025-03-18

**Authors:** Xiaoting Zhang, Huaan Li, Lu Liu, Yanzhen Song, Lishan Zhang, Jiajun Miao, Jiamiao Jiang, Hao Tian, Chang Liu, Fei Peng, Yingfeng Tu

**Affiliations:** a NMPA Key Laboratory for Research and Evaluation of Drug Metabolism & Guangdong Provincial Key Laboratory of New Drug Screening, School of Pharmaceutical Sciences, Southern Medical University, Guangzhou 510515, China; b Guangdong Provincial Key Laboratory for Research and Evaluation of Pharmaceutical Preparations & Guangdong Provincial Engineering Center of Topical Precise Drug Delivery System, Center for Drug Research and Development, Guangdong Pharmaceutical University, Guangzhou 510006, China; c Sport Science College, Beijing Sport University, Beijing 100091, China; d School of Materials Science and Engineering, Sun Yat-Sen University, Guangzhou 510275, China

**Keywords:** Microrobots, Biological orthogonal reaction, Biofilms, *Chlamydomonas reinhardtii*, Alginate lyase, Microalgae, Antibacterial therapy, Photodynamic therapy

## Abstract

Bacterial biofilms can make traditional antibiotics impenetrable and even promote the development of antibiotic-resistant strains. Therefore, non-antibiotic strategies to effectively penetrate and eradicate the formed biofilms are urgently needed. Here, we demonstrate the development of self-propelled biohybrid microrobots that can enhance the degradation and penetration effects for *Pseudomonas aeruginosa* biofilms in minimally invasive strategy. The biohybrid microrobots (CR@Alg) are constructed by surface modification of *Chlamydomonas reinhardtii* (CR) microalgae with alginate lyase (Alg) *via* biological orthogonal reaction. By degrading the biofilm components, the number of CR@Alg microrobots with fast-moving capability penetrating the biofilm increases by around 2.4-fold compared to that of microalgae. Massive reactive oxygen species are subsequently generated under laser irradiation due to the presence of chlorophyll, inherent photosensitizers of microalgae, thus triggering photodynamic therapy (PDT) to combat bacteria. Our algae-based microrobots with superior biocompatibility eliminate biofilm-infections efficiently and tend to suppress the inflammatory response *in vivo*, showing huge promise for the active treatment of biofilm-associated infections.

## Graphical abstract

Self-propelled CR@Alg microrobots penetrate and eliminate medical device-associated biofilms by disrupting the three-dimensional structure of the matrix and generating ROS to damage bacteria.

**Figure:**
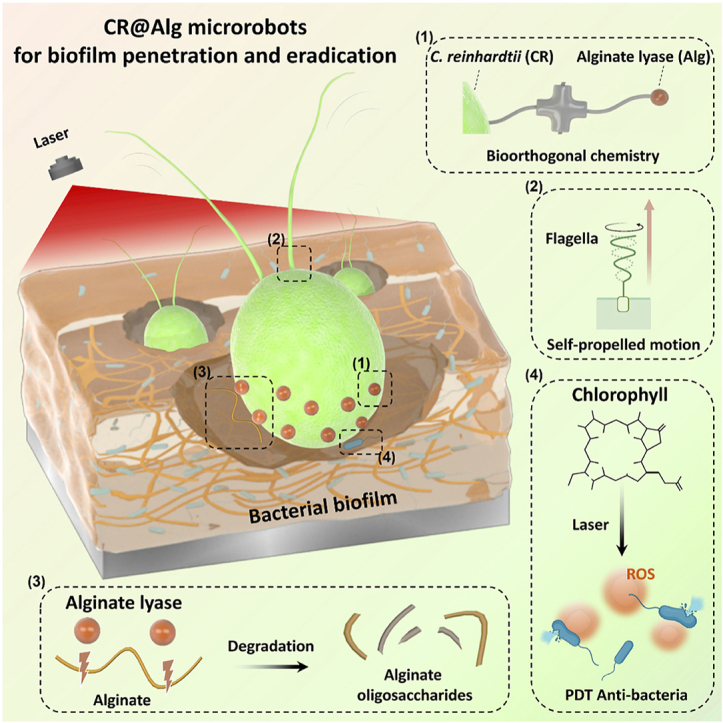

## 1 Introduction

With the widespread use of various medical devices (central venous catheters, urinary catheters, etc.), the safety issue of invasive treatment becomes increasingly important. The surfaces of medical devices that have been the foci of device-related infections show the presence of large numbers of slime-encased bacteria^1^^,^^2^. Biofilm-associated microorganisms generally cause a great deal of infections^3^. Over 600 million people annually suffer from catheter-related bloodstream infections (CRBSI), catheter-associated urinary tract infections (CAUTI), and ventilator-associated pneumonia (VAP)^4^^,^^5^. Also, non-device-related chronic biofilm diseases were commonly seen in patients with cystic fibrosis^6^, chronic wound infections^7^, and chronic prostatitis^8^.

Biofilms are three-dimensional multicellular communities with extracellular matrix (ECM) including exopolysaccharide, extracellular DNA (eDNA), RNA, lipids, and extracellular membrane vesicles. These components enable bacteria to adhere irreversibly to indwell medical instruments and protect microorganisms against environmental assaults^6^^,^^9^^,^^10^. As a hyper secreted exopolysaccharide of ECM, alginate contributes to the formation of structured and heterogeneous biofilms^6^. The functions of alginate include facilitating cell adhesion, aggregation, cell-to-cell, and cell-to-biofilm connections, supporting biofilm development by providing strength and rigidity through calcium chelation^11^^,^^12^. Therefore, biofilms on medical devices allow bacteria to become resistant to antibiotic treatment, block the flow of urine or bloodstream through the catheter, and prevent eradication from innate immune cells, resulting in persistent infection, infectious complications, and devastating device malfunction (intravascular catheter dysfunction, biliary tube obstruction, crystalline encrustations on urinary stents, etc.) in clinical practice, which required surgical debridement and device removal^8^.

A recent study has demonstrated that alginate lyase can efficiently degrade the secreted alginate of *Pseudomonas aeruginosa* (*P. aeruginosa*) by breaking the glycosidic bond through a *β*-elimination reaction, resulting in the generation of oligomers with 4-deoxy-L-erythro-hex-4-enepyranosyluronate acid at the nonreducing end^6^. Therefore, alginate lyase has the potential to disrupt the biofilm architecture, enhance antibiotic distribution, and promote existing therapy effects by hydrolyzing the negatively charged alginate^11^^,^^13^. Furthermore, the degradation products of alginate, reported as alginate oligosaccharides (AOs), have been found they show various physiological activities, including immunomodulatory, antihypertensive, and anticoagulant properties^14^. However, biofilm-associated infections need a therapeutic strategy with penetrating properties, and alginate lyase can only damage the surface of biofilms and subsequently show a potential anti-bacterial effect. Wu et al.^15^ designed a pH-sensitive system, which exhibited better biofilm penetration and eradication *via* electrostatic interactions. Additionally, Mayorga-Martinez et al.^16^ used aqua sperm micromotors with rapid velocity and snake-like undulatory locomotion for biofilm damage, highlighting the importance of a drug's capability to infiltrate the biofilms for effective eradication.

Compared with traditional passive particle systems, micro/nanomotors, especially for biohybrid motors/robots received strong attention in various fields, due to their ability for precise navigation and tissue permeability^17^^,^^18^. Based on living systems (including bacteria, microalgae, sperms, etc.), biohybrid microrobots have been widely applied in cargo transportation^19^, wireless actuation^20^, anti-bacterial therapy^21^, tumor-targeting^22^ and various other aspects in biomedicine. Notably, biohybrid micromotors based on *Chlamydomonas reinhardtii* (*C. reinhardtii*) microalgae have a promising prospect in biomedicine, since showing much more adaptability to the complicated microenvironment^23^, minimal invasiveness^24^, and good biocompatibility^25^. Due to the existing eukaryotic flagella (≈12 μm long), as linear motors containing an inner core of microtubules and associated motor proteins (the axoneme), *C. reinhardtii* demonstrate efficient locomotion for versatility in diverse aqueous environments without any toxic fuel^26^. Also, the cell wall of *C. reinhardtii*, provides facile surface modification, and the amino-terminal residue and structure (such as 4-hydroxyproline (4-HP)-rich glycopeptides)^27^, ^28^, ^29^ can be exploited to immobilize cargoes such as polypeptides^26^, liposomes^30^, and other materials *via* combinations of covalent or noncovalent interactions^26^. Furthermore, the abundant chlorophyll in *C. reinhardtii*, as a natural photosensitizer, can generate reactive oxygen species (ROS) under specific laser irradiation^31^.

Because of quorum sensing^32^, a cell-to-cell communication system used by bacteria to collaborate, reproduce, and protect from biofilms, bacteria can eventually evolve to exhibit antibiotic resistance to new drugs^33^. Nevertheless, among the non-antibiotic treatments, photodynamic therapy (PDT) is minimally invasive, site-specific, and can be used to eradicate various bacteria, even those resistant to antibiotics^34^. PDT utilizes photosensitizers and lasers to trigger ROS, such as singlet oxygen (^1^O_2_) and superoxide anion (O^2−^). ROS disrupts the eDNA, protein, and membrane assembly directly, leading to bacteria suffering oxidative stress and death^35^. Studies showed that the infrared light (wavelength range: 495–630 nm) of PDT can penetrate 3–6 mm depth underneath the skin, covering the epidermis (100 μm thick) and dermis (1–4 mm thick) to fight bacterial infections^36^. Therefore, it is necessary to preserve the viability of *C. reinhardtii* for multiple applications^30^. Thus, the construction of biohybrid micromotors using bioactive substances directly without the usage of synthetic materials as cargo becomes increasingly focused, such as polypeptides and enzymes^19^. Hence, to preserve the respective activities of both alginate lyase and *C. reinhardtii* as much as possible, the bioorthogonal method without the requirement of copper catalysis is used for the construction of microrobots, in accordance with the idea of click chemistry, which is a technique for efficiently joining molecules together quickly and without unwanted by-products^37^. Streptavidin-biotin cross-linking, a type of bioorthogonal method, is commonly used in tissue engineering^38^ and has become one of the strongest known noncovalent interactions in nature, such as reinforcing the attachment for cargo delivery, tracking, and signal enhancement^19^^,^^39^^,^^40^.

**Scheme 1:**
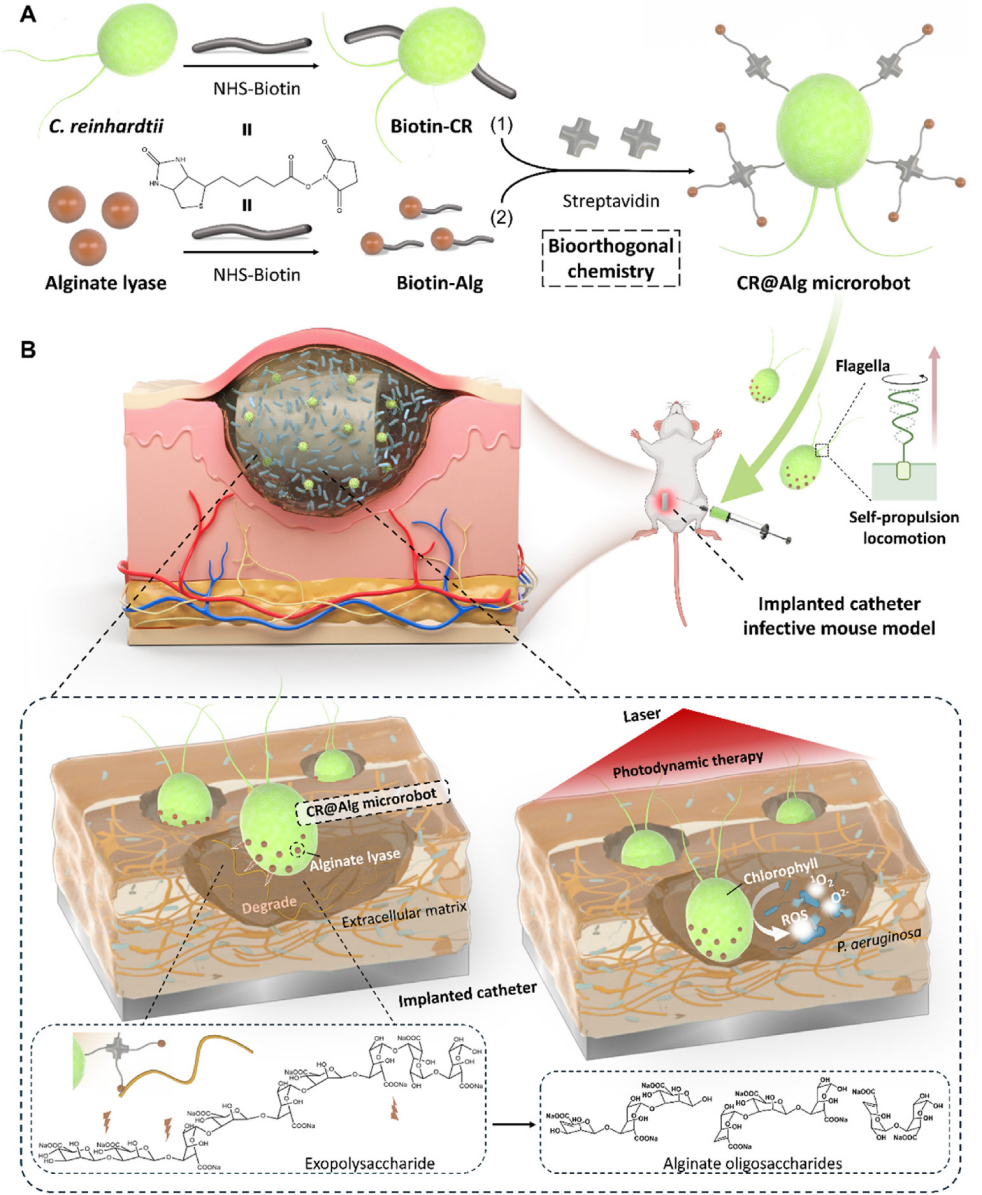
Schematic illustration presenting the fabrication and application of CR@Alg microrobots. (A) Schematic of CR@Alg microrobots assembly *via* biological orthogonal reaction. (B) Schematic of the mechanism of self-propelled microrobots exhibiting efficient biofilm penetration and antibacterial treatment upon 638 nm laser irradiation on an implanted catheter infective mouse model.

Herein, we developed bioinspired microrobots consisting of enzyme-immobilized microalgae to facilitate deep biofilm penetration and treat bacterial infections. As illustrated in Scheme 1, the biohybrid microrobot was based on *C. reinhardtii* modified with active alginate lyase *via* the biotin-streptavidin-biotin self-assembly system (denoted as ‘CR@Alg microrobots’). Our self-propelled microrobots exhibit efficient biofilm penetration due to the alginate degradation with alginate lyase creating surface shortfalls on biofilms so that the depth of biofilms was much easier to be penetrated by the microrobots. Since ROS, generated by CR@Alg microrobots, acts as strong oxidants to damage micro-organisms directly, CR@Alg microrobots showed stable killing efficiency against *P. aeruginosa* in both of *in vitro* and *in vivo* models. The inherent properties of environmentally friendly microalgae are ingeniously combined with active alginate lyase to produce a self-propelled microrobot capable of biofilm penetration, providing a minimally invasive drug-free therapy to eradicate biofilms on implanted medical instruments *in vivo*.

## 2 Materials and methods

### 2.1 Fabrication of CR@Alg microrobots

*C. reinhardtii* were obtained from the Institute of Hydrobiology, Chinese Academy of Science (FACHB-355, Wuhan, China), and cultivated in Tris-acetate-phosphate (TAP) medium (Phyto Technology, T8224-10L, Lenexa, USA), at 23 °C under the cycles of 12 h sunlight and 12 h dark. *C. reinhardtii* were centrifuged at 500×*g* for 3 min (DLAB D3024R, Beijing, China), washed with DI water to remove any residual TAP medium, and subsequently resuspended in DI water for optical density (OD) for microalgae at the wavelength of 660 nm. Then, 1 mL of 6.0 × 10^7^ *C. reinhardtii* were incubated with Sulfo–NHS–Biotin (1 mg/mL, Thermo Fisher Scientific, A39256, Shanghai, China) for 1 h at 25 °C. The resulting biotin-modified-*C. reinhardtii* (biotin-CR) were centrifuged (700×*g*, 3 min), washed with DI water and then redispersed in 500 μL of DI water, followed by dropwise addition of streptavidin (0.5 mg in DI water, APExBIO Technology LLC., B7921, Houston, USA) and subsequent reaction for 1 h at 25 °C to allow streptavidin conjugated to biotin-CR. After centrifugation at 700×*g* for 3 min and washing with DI water, streptavidin-biotin-CR were prepared. In parallel, 1 mg/mL of alginate lyase (Sigma–Aldrich, A1603, Shanghai, China) was incubated with Sulfo–NHS–Biotin (1 mg/mL), and the resulting solution was stirred for 1 h at 25 °C. The obtained biotin-modified-alginate lyase (biotin-Alg) were concentrated by filtration (10 kDa molecular weight cut-off, Amicon Ultra 0.5 mL Filters, Merck Millipore, UFC5010, Massachusetts, USA) at 6000×*g* for 15 min and washed three times with DI water to remove excess NHS ester. CR@Alg microrobots were then synthesized by mixing streptavidin-biotin-CR with biotin-Alg for 1 h incubation *via* bioorthogonal chemistry. Scanning electron microscope (SEM) images were recorded on a Phenom emission scanning electron microscope (Nikon, Phenom ProX, Tokyo, Japan). Alginate lyase conjugating results were analyzed by flow cytometer (Becton Dickinson, LSR Fortessa X-20, New Jersey, USA).

### 2.2 Motion analysis of CR@Alg microrobots

The motion of CR@Alg microrobots and *C. reinhardtii* were analyzed in Phosphate buffers (PBS), TAP, DI water (H_2_O) and Tryptic Soy Polmyxin Brot Base (TSB) medium (Merck Millipore, 22092, Massachusetts, USA) in Petri dishes. The inverted fluorescence microscope (Nikon, Ti2-A, Tokyo, Japan) was used to record the motion of microrobots. Time-lapse images were recorded (time interval between each frame *Δ*T = 200 ms). The velocity of CR@Alg microrobots was evaluated at 0 and 24 h. Tracking image sequences were analyzed with the Image J plugin manual tracking.

### 2.3 Alginate lyase hydrolytic activity assay

Enzyme activity was performed as the method reported in the previous study^41^. Briefly, 400 μL of sodium alginate (Sigma–Aldrich, 180947, Shanghai, China) solution (10 mg/mL) in PBS (pH 6.5) was incubated with 100 μL alginate lyase (20 μg/mL), CR@Alg (5.0 × 10^6^ algae/mL), *C. reinhardtii* (5.0 × 10^6^ algae/mL) and PBS for 10 min at 37 °C. Finally, 40 μL of 10 mol/L NaOH was added to stop the reaction and absorbance at 235 nm was measured on a spectrophotometer (SHIMADZU, UV-2600, Tokyo, Japan).

### 2.4 ROS detection

The ROS generation capability of CR@Alg microrobots upon laser irradiation was then investigated using 2′,7′-dichlorodihydrofluorescein diacetate (DCFH-DA, Beyotime Biotechnology, S0033S, Shanghai, China). It was reported that in the presence of ROS, the transferred 2′,7′-dichlorofluorescein (DCFH) by DCFH-DA would be rapidly oxidized and form fluorescent molecule (dichlorofluorescein, DCF). DCFH solution (5 μmol/L) and *C. reinhardtii* (5.0 × 10^6^ algae/mL) were placed in a 1.5 mL EP tube, exposed to 638 nm laser irradiation (0.6 W/cm^2^) for varied time intervals (0, 10, 20, 30, 40, 50, 60, 70, 80, 90 min). The ROS generation as determined by DCF (excitation, *E*_x_: 488 nm; emission, *E*_m_: 525 nm), was recorded as fluorescence spectrum using fluorescence spectroscopy (SHIMADZU, RF-6000, Tokyo, Japan). DCFH in the group of *C. reinhardtii* (5.0 × 10^6^ algae/mL) was lasered by 638 nm laser irradiation for 30 min, for varied power density (0, 0.1, 0.3, 0.6, 0.9, 1.2 W/cm^2^), record the fluorescence spectrum using fluorescence spectroscopy. DCFH in the groups of *C. reinhardtii*, CR@Alg (5.0 × 10^6^ algae/mL), PBS medium, and alginate lyase were lasered by 638 nm laser irradiation (0.6 W/cm^2^, 30 min) record the fluorescence spectrum using fluorescence spectroscopy (SHIMADZU).

### 2.5 DCFH-DA staining fluorescent images of P. aeruginosa

*Pseudomonas aeruginosa* (*P. aeruginosa,* Guangdong Microbial Culture Collection Center, ATCC 27853, Guangzhou, China) was first streaked onto a Luria broth (LB, Sigma–Aldrich, L3022, Shanghai, China) agar plate and cultured overnight at 37 °C. A single colony was inoculated in 100 mL LB medium in a conical bottle and further cultured for 10 h at 37 °C on a shaker 200 rpm.

*P. aeruginosa* (1.0 × 10^6^ CFU/mL) suspensions were placed in 96-well plates, and treated with TSB medium, 638 nm laser irradiation (0.6 W/cm^2^, 30 min), *C. reinhardtii* (dark), *C. reinhardtii* + Laser, CR@Alg (dark), CR@Alg + Laser, *C. reinhardtii* and CR@Alg (1.0 × 10^7^ algae/mL). At 2.5 h after treatment, the bacterial cells were collected and stained with DCFH-DA at 37 °C for 80 min, followed by DAPI staining (Sigma–Aldrich, MBD0020, Shanghai, China). Then, bacterial cells were washed with PBS and the images were captured by a confocal laser microscope (Olympus, FV3000, Tokyo, Japan). “L” represents “under 0.6 W/cm^2^ power density laser irradiation for 30 min”.

### 2.6 3D fluorescent images of biofilm penetration

*P. aeruginosa* was washed with PBS, and then resuspended in TSB medium to a final concentration of 5.0 × 10^8^ CFU/mL, followed by being placed in 96-well plates at 37 °C for 48 h to allow the formation of biofilms. The biofilms were treated with *C. reinhardtii* (dark), *C. reinhardtii* + Laser, CR@Alg microrobots (dark), CR@Alg microrobots + Laser, *C. reinhardtii* and CR@Alg (5.0 × 10^6^ algae/mL), laser groups were under 0.6 W/cm^2^ power density laser irradiation for 30 min, respectively. At 1.5 h after treatment respectively, the amount of CR@Alg microrobots that remained in the biofilms was observed under a fluorescence confocal microscope (Nikon), to evaluate their penetrative effect. The 3D fluorescent images of *C. reinhardtii* in the red channel and biofilms with DAPI staining in the blue channel.

### 2.7 3D fluorescent images biofilm eradication

*P. aeruginosa* (200 μL, 1.0 × 10^8^ CFU/mL per well) was incubated in TSB medium and placed in 96-well plates at 37 °C for 48 h to allow the formation of biofilms. The biofilms were treated with TSB medium, 638 nm laser irradiation (0.6 W/cm^2^, 30 min), *C. reinhardtii* (dark), *C. reinhardtii* + Laser, CR@Alg (dark), CR@Alg + Laser, *C. reinhardtii* and CR@Alg (5.0 × 10^6^ algae/mL), respectively. At 3 h after treatment, the biofilms were washed with PBS gently, and then the bacteria were stained with Hoechst 33342 (Thermo Fisher Scientific, H1399, Shanghai, China). Finally, the destructive conditions within the biofilms and their 3D fluorescent images were taken on an inverted fluorescence microscope (Nikon).

### 2.8 Crystal violet staining measurements

*P. aeruginosa* biofilms formed in 96-well plates were exposed to *C. reinhardtii* or CR@Alg (density of 0, 5.0 × 10^5^, 1.0 × 10^6^, 5.0 × 10^6^, 1.0 × 10^7^ algae/mL), then treated with 638 nm laser irradiation (0.6 W/cm^2^, 30 min) or in the dark after 2 h of incubation. Followed by washing with PBS to remove the disintegrated biofilms after treating with *C. reinhardtii* and CR@Alg for 24 h, the biofilms were fixed with absolute methanol (150 μL) for 15 min. After discarding the absolute methanol and thoroughly drying, crystal violet (Beyotime Biotechnology, C0121, Shanghai, China) solutions (100 μL, *w/w* 1% in PBS) were added to each well for 10 min to stain the biofilms. After staining, the biofilms were washed with PBS to remove the excess crystal violet and thoroughly dried. The stained biofilms were dissolved by adding acetic acid (200 μL, *v/v*, 33% in PBS) and measured at 570 nm on a microplate reader (BioTek, 800 TS, Vermont, USA).

### 2.9 Animal experiment of the implanted catheter infective model

BALB/c mice (6 weeks, female) were supplied by Laboratory Animal Center of Southern Medical University. All experimental protocols were carried out under the guidelines approved by the Institutional Animal Care and Use Committee (IACUC) of Southern Medical University (permit number: SMUL202312009).

To investigate the *in vivo* therapeutic effects of CR@Alg microrobots, subcutaneous murine *P. aeruginosa*-infected implanted models in BALB/c mice were used. To form catheters with adhering biofilms, commercial vacuum catheters (B. Braun, Venofix® A, Melsungen, Germany) were cut into 10 mm segments, sterilized with 75% ethanol, and then 1 mL of TSB medium containing 1.0 × 10^8^ CFU/mL of *P. aeruginosa* were incubated with each 10 mm-catheter at 37 °C for 48 h. The catheters washed by PBS were implanted in the inner thigh of mice. After 24 h, all the mice were randomly divided into 6 groups (*n* = 4), treated with blank PBS without irradiation, only 638 nm laser irradiation (0.6 W/cm^2^, 30 min), *C. reinhardtii* (dark), *C. reinhardtii* + Laser, CR@Alg (dark), CR@Alg + Laser groups. 50 μL *C. reinhardtii* or CR@Alg (1.0 × 10^7^ algae/mL) was injected to the infectious sites, including the interior and the surrounding of the implanted catheters so that the infected areas were fully infiltrated. After 1 h, when the wound heals, caused by the puncture of the syringe used to administer the drug, the infectious sites were treated with 638 nm laser irradiation (0.6 W/cm^2^, 30 min). The treatment was proceeded 3 times on Days 1, 3, and 5 after implantation surgery. On Days 2, 4, and 7, the mice were euthanatized, in parallel the implanted catheters and tissues around the infectious sites were obtained.

### 2.10 Bacteria counting by agar plate dilution method

To evaluate the eradication of biofilm-infection, bacteria of the recollected catheters from the infectious sites were counted by the typical agar plate dilution method. In detail, the catheters were completely immersed in 4 mL physiological saline in centrifuge tubes and sonicated for 30 min under a water bath to make the bacteria fully into suspension. Then the suspension was diluted and spread onto LB agar plates. After incubation for 20 h at 37 °C, the bacterial plaques on the plate were counted.

### 2.11 Quantitative analysis of the level of inflammatory cytokines

The anti-inflammatory effect of CR@Alg was assessed by ELISA assays. Specifically, 1 mm^3^ infectious tissues from mice were separated and weighted. After being immersed in PBS (1:9), and thoroughly mashed under an ice bath, the tissues were centrifugated (900×*g*, 20 min, 4 °C). Then the supernatants were collected to quantify the concentration of proinflammatory cytokines IL-1*β*, IL-6, and TNF-*α* by using corresponding ELISA kits. IL-1*β* ELISA kit was purchased from Elabscience (E-EL-M0037c, Wuhan, China) ELISA kits for IL-6 and TNF-*α* were supplied by Dakewe Biotechnology (1210602, 1217202, Shenzhen, China). Experiments were performed three times.

### 2.12 Statistical analysis

Data analysis was carried out *via* Student's *t*-test (two-tailed), and Image J (1.52a). Data was expressed as means ± standard deviation (SD) from several separate experiments. The asterisk was considered as statistical significance: ∗*P* < 0.05, ∗∗*P* < 0.01, ∗∗∗*P* < 0.001, ∗∗∗∗*P* < 0.0001.

## 3 Results and discussion

### 3.1 Fabrication and characterization of CR@Alg microrobots

**Figure 1:**
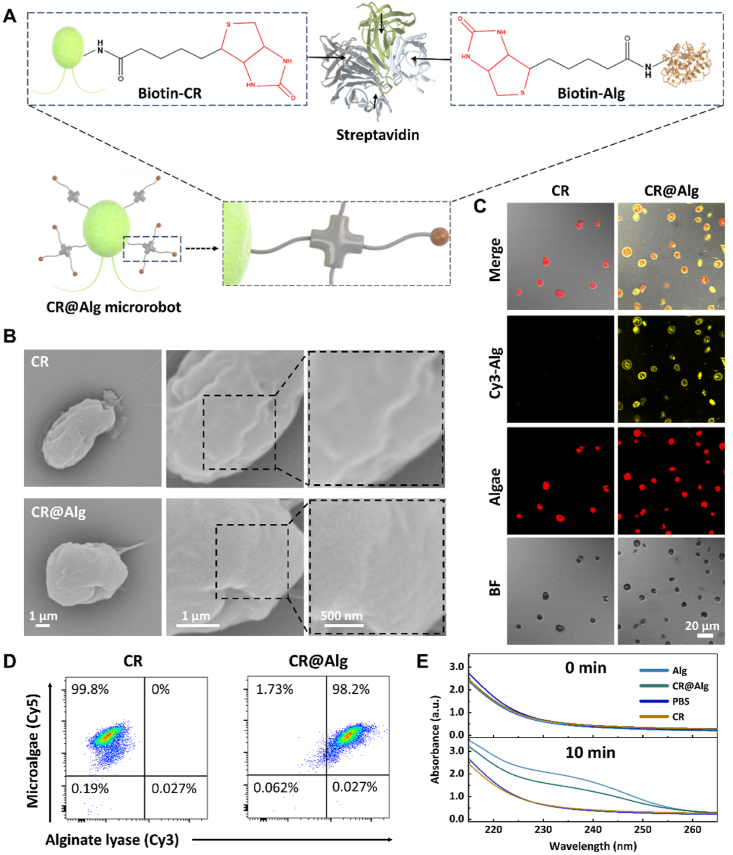
Characterization of alginate lyase-functionalized *C. reinhardtii* microrobots (referred to as CR@Alg microrobots). (A) Schematic illustration presenting biological orthogonal reaction. (B) SEM images of *C. reinhardtii* and CR@Alg microrobot. (C) Fluorescent images of CR@Alg microrobots. Cy3-labeled alginate lyase (Cy3-Alg, yellow channel) was conjugated to *C. reinhardtii* with autofluorescence (*E*_x_: 650 nm, red channel). (D) Flow cytometry analysis of *C. reinhardtii* (Cy5 channel) before and after functionalization with Cy3-labeled alginate lyase. (E) Alginate hydrolytic activity of CR@Alg microrobots at body temperature 37 °C, represented as the increased absorbance at 235 nm over 10 min.

As the main component of microrobots, *C. reinhardtii* were first cultured in TAP medium and exhibited uniform ovoid morphology with 8.7 ± 0.29 μm average diameter (Supporting Information Fig. S1). As a typical biological orthogonal reaction, biotin-streptavidin-biotin system was utilized to introduce alginate lyase onto the surface of *C. reinhardtii* (Fig. 1A). In detail, Sulfo–NHS–biotin was first conjugated to alginate lyase and *C. reinhardtii* respectively, *via* the N-hydroxysuccinimide (NHS) ester reaction. As a mediator, each streptavidin tetramer has four independent biotin-binding sites. One biotin of biotin-modified *C. reinhardtii* (CR-biotin) was bound to one active binding site of streptavidin *via* strong noncovalent interaction. After biotin-modified alginate lyase (Alg-biotin) conjugating to other active biotin-binding sites of streptavidin-biotin-CR, CR@Alg microrobots were then successfully constructed. Compared to the bare *C. reinhardtii* with a smooth surface, the immobilized alginate lyase could be clearly observed on the surface of CR@Alg microrobots with roughness as shown in SEM images (Fig. 1B). Fluorescent Cy3-NHS ester was then used to label the anchored alginate lyase on *C. reinhardtii*. As illustrated in Fig. 1C, Cy3-labeled-alginate lyase emitted a strong fluorescent signal in yellow channel and was also colocalized with those of the autofluorescence of chlorophyll from *C. reinhardtii* in a red round shape. Then, to further perform the quantitative analysis, flow cytometry results were showed in Fig. 1D. 98.2% of the *C. reinhardtii* population emitted fluorescent signals in the Cy3 channel, indicating that a considerable amount of *C. reinhardtii* was modified with Cy3-labeled-alginate lyase. In Supporting Information Fig. S2, CR@Alg microrobots showed corresponding fluorescent peaks of Cy3-labeled-alginate lyase (Ex:550 nm, Em:570 nm), indicating the successful introduction of alginate lyase onto *C. reinhardtii via* the bioorthogonal chemistry.

Because of the modification with Sulfo–NHS–biotin *via* the biorthogonal method, alginate lyase retained the capacity of alginate degradation, normally resulting in dispersing the three-dimensional structure of the bacterial biofilm matrix. Therefore, the enzyme activity of alginate lyase was then evaluated according to the previous report^41^. As shown in Fig. 1E, around 74.9 ± 1.9 % of alginate lyase enzymatic activity was reserved after bioorthogonal reaction, indicating that the introduced alginate lyase with relatively high enzyme activity is possible to trigger the efficient degradation of alginate.

### 3.2 Motion analysis of CR@Alg microrobots

**Figure 2:**
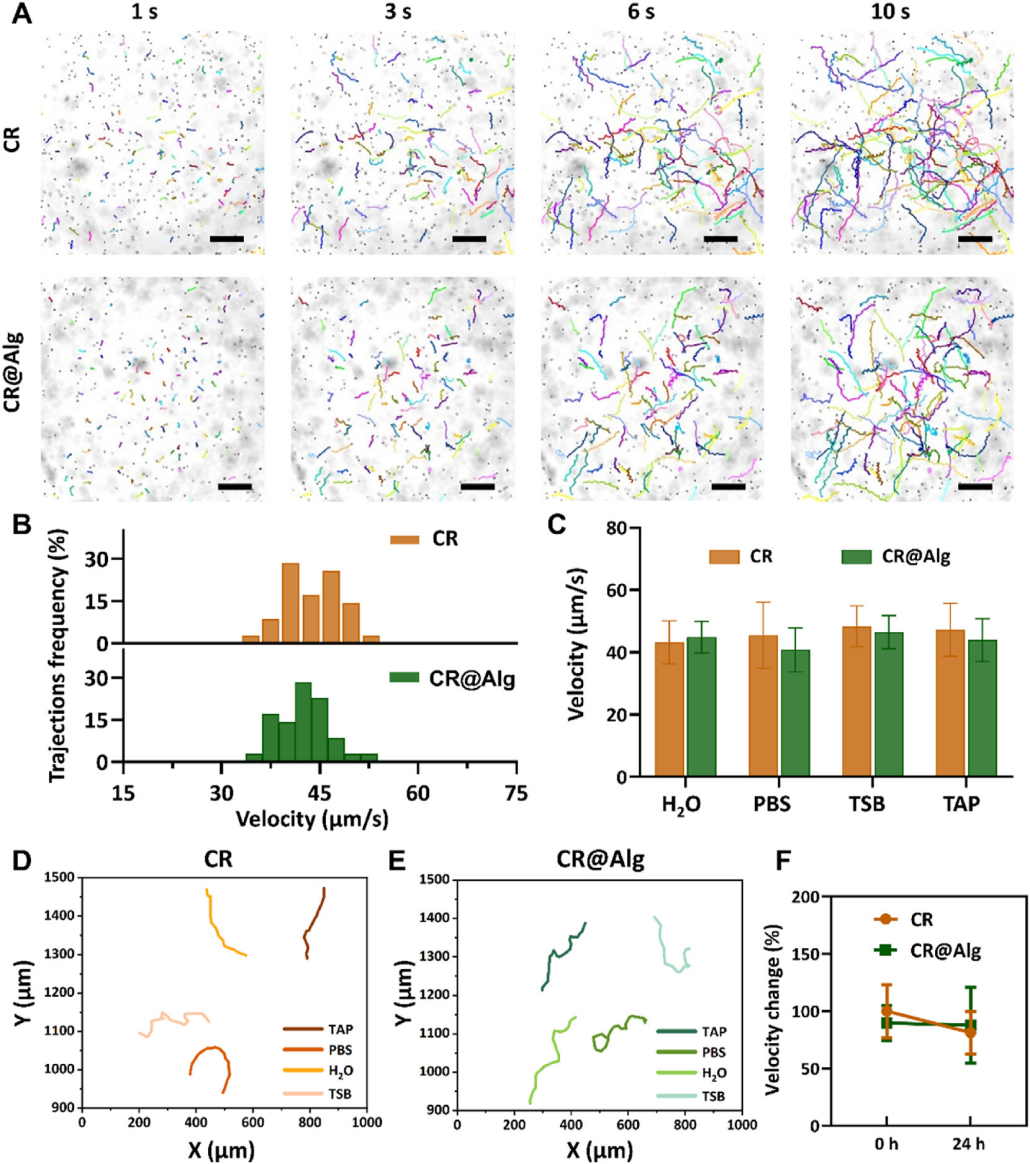
The motion of CR@Alg microrobots. (A) Time-lapse images showing the representative trajectories, data are presented as mean ± SD (*n* = 100, scale bar = 200 μm) and (B) mean velocity distribution of *C. reinhardtii* and CR@Alg microrobots in PBS at 37 °C. (C) The velocity of *C. reinhardtii* and CR@Alg microrobots in H_2_O, PBS, TSB, and TAP media at 37 °C, respectively. (D) and (E) Representative tracking trajectories of *C. reinhardtii* and CR@Alg microrobots in TAP, PBS, H_2_O, and TSB media. (F) The velocity change of CR@Alg microrobots, cultured for 24 h in PBS at body temperature (37 °C).

The unicellular photosynthetic *C. reinhardtii* normally use two eukaryotic flagella (12 μm long) for the locomotion and biological propulsion^42^. The flagellum consists of a distinctive microtubule structure^43^, controlled by each interlocking subunit *via* torque transmission to deform the shape actively. As puller microswimmers, microalgae presents breaststroke propelling motility, drawing the front site of fluid to the cell surface for the construction of a nutrient flux^42^. In this study, the motion of CR@Alg microrobots was recorded in PBS. As shown in Fig. 2A, time-lapse images and representative trajectories of *C. reinhardtii* and CR@Alg microrobots over 1, 3, 6 and 10 s intervals were clearly presented and demonstrated similar trends in their motion. The corresponding mean velocity distribution and values of *C. reinhardtii* and CR@Alg microrobots were measured to be 45.5 ± 10.6 and 40.8 ± 7.0 μm/s in PBS, respectively (Fig. 2B). Similar results of velocity and trajectories were obtained for CR@Alg microrobots and *C. reinhardtii* swimming in different media (H_2_O, PBS, TSB, and TAP media), reflecting that the motion capability of algae-based microrobots was barely affected after alginate lyase functionalization and showing adaptivity under diverse environments (Fig. 2C‒E). Our CR@Alg microrobots maintained a steady speed and achieved distances of 4.69 body lengths per second, providing an approach to attacking biofilms at a wider and deeper range.

Then, we further recorded the long-term motion behaviors of CR@Alg microrobots. Notably, Fig. 2F showed the velocity change in the motion of CR@Alg microrobots and 90% of microrobots preserved activity with steady velocity after 24 h of motility in PBS, indicating the great adaptivity of microalgae without TAP medium for a longer time and becoming viable for *in vivo* conditions. These results verified the negligible cytotoxicity of alginate lyase functionalization on *C. reinhardtii* and they still displayed fast movement capability. Also, it has been indicated that the biotin-streptavidin-biotin system, as a typical biological orthogonal reaction, does not compromise the intrinsic mobility of unmodified microalgae^44^. Furthermore, as shown in Supporting Information Fig. S3A‒S3C, the similar tracking trajectories were recorded and the corresponding mean velocity of CR@Alg microrobots after 30 min 638 nm laser irradiation with 0.6 W/cm^2^ were measured to be 41.96 ± 3.5 μm/s in PBS, reflecting that the motion capability of CR@Alg microrobots were barely affected by the generated ROS during PDT.

### 3.3 *In vitro* ROS generation

**Figure 3:**
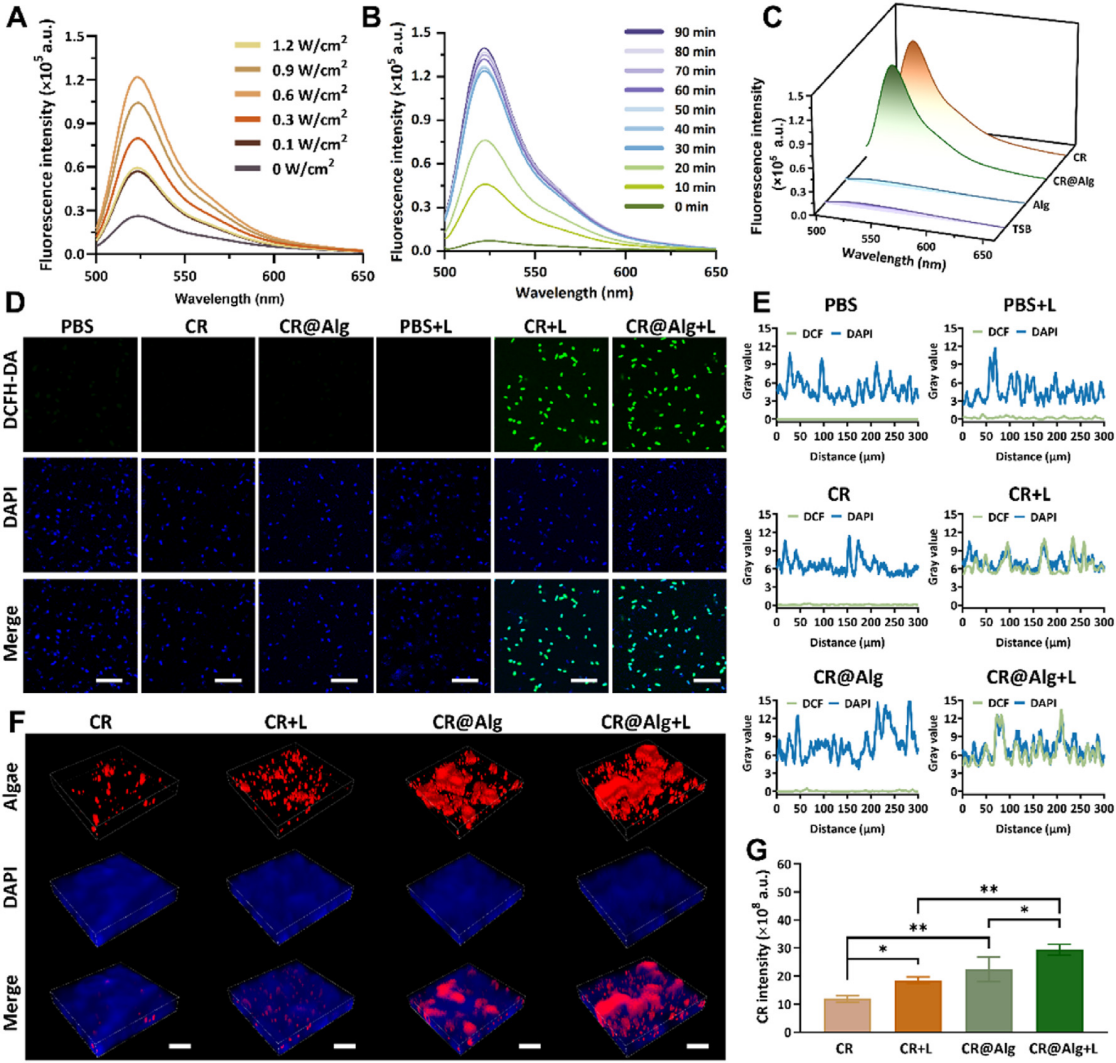
ROS-producing activity. (A) Fluorescence spectra of DCF in *C. reinhardtii* lasered by 638 nm laser irradiation (30 min) for varied power density. (B) Fluorescence spectra of DCF in *C. reinhardtii* lasered by 638 nm laser irradiation (0.6 W/cm^2^) for varied time intervals. (C) Fluorescence spectra of DCF in *C. reinhardtii* and CR@Alg, TSB medium, and alginate lyase lasered by 638 nm laser irradiation, 0.6 W/cm^2^, 30 min. (D) Fluorescent images of DCFH-DA (green) and DAPI (blue) staining of *P. aeruginosa* cells after different treatments (scale bar = 10 μm). (E) The grey value of DCF across the bacteria and DAPI colocalization. (F) 3D fluorescence images of *C. reinhardtii*, *C. reinhardtii* + Laser, CR@Alg, and CR@Alg + Laser to evaluate their penetrative effect of *P. aeruginosa* biofilms (DAPI, blue). Autofluorescence of *C. reinhardtii* chloroplast in the red channel (scale bar = 100 μm). (G) Quantification of *C. reinhardtii* and CR@Alg in biofilms. Data are presented as mean ± SD (*n* = 4). ∗*P* < 0.05, ∗∗*P* < 0.01. “L” represents “Laser”.

Since *C. reinhardtii* contain natural photosensitizers chlorophyll, their excellent ROS-producing capacity can be triggered by laser irradiation of particular wavelengths^45^. By promoting the ROS level to induce oxidative stress, the eDNA and protein in ECM can be disrupted directly and the bacterial innate redox homeostasis can be disturbed^35^. To investigate the ROS production property of CR@Alg, the level of ROS was determined by the fluorescence intensity change of a ROS probe, DCFH-DA. After incubating with sodium hydroxide, DCFH-DA was transformed to nonfluorescent DCFH, which can be oxidized by ROS to become a fluorescent DCF molecule rapidly^46^. *C. reinhardtii* (5 × 10^6^ algae/mL) were incubated with DCFH under a 638 nm laser irradiation with varied power densities, followed by measuring the fluorescence intensity of DCF at 530 nm that represented the level of ROS. As shown in Fig. 3A, the fluorescence intensity of DCF increased to 1.2 × 10^5^ a.u. within 30 min triggered by *C. reinhardtii* under the irradiation of 638 nm laser (0.6 W/cm^2^, Supporting Information Fig. S4A). However, it was found that the ROS production property of *C. reinhardtii* cannot be strengthened by increasing the power density of 638 nm laser irradiation. Subsequently, the fluorescence spectra showed the progression of ROS generation by *C. reinhardtii* over time under 638 nm laser irradiation (0.6 W/cm^2^). As shown in Fig. 3B and Supporting Information Fig. S4B, an obvious enhancement of DCF fluorescence intensity triggered by *C. reinhardtii* upon the laser irradiation (0.6 W/cm^2^) between 0 and 30 min was clearly observed, indicating massive production of ROS. Whereas the fluorescence intensity of *C. reinhardtii* under irradiation between 30 and 90 min remained at a slight increase.

Compared with the fluorescence spectra and the curve of *C. reinhardtii*, CR@Alg microrobots still performed great talent for ROS production (Fig. 3C and Supporting Information Fig. S4C). This was consistent with the activity of *C. reinhardtii* before modification of alginate lyase. Besides, the fluorescence intensity of TSB medium and single alginate lyase remained at a low level. To further investigate ROS produced by CR@Alg microrobots in biofilms, we detected the level of ROS by DCFH-DA staining in different groups. As depicted in Fig. 3D, the fluorescent images demonstrated that strong signals with green fluorescence were captured in *C. reinhardtii* + Laser and CR@Alg + Laser groups. Barely fluorescent signal was observed in CR@Alg group without laser treatment, and almost no significant fluorescent signal for PBS and PBS + Laser group because of *C. reinhardtii* absence. In addition, Image J was then utilized to quantitatively analyze DCF fluorescence intensity of *P. aeruginosa* in different groups (Supporting Information Fig. S5), indicating that the ROS level was obviously increased. The grey value of DCF signal in bacteria and DAPI signal colocalization analyzation (Fig. 3E) showed that the DCF fluorescent signal emitted by the ROS were almost at the same position as DAPI signal, proving that the ROS indeed diffused into bacterial cells. The fluorescence intensity of CR@Alg + Laser and *C. reinhardtii* + Laser groups were both higher than 160 a.u., while the fluorescence intensity of PBS, *C. reinhardtii*, and CR@Alg groups were all lower than 25 a.u., which might imply the antibacterial effect due to the high photosensitive activity of ROS production of *C. reinhardtii* upon the laser irradiation. The consistent results of DCF fluorescent signal can also be observed in *P. aeruginosa* biofilms (Supporting Information Fig. S6A and S6B). Notably, because of adaptive locomotion and long lifespan, the challenges of short diffusion distance and limited lifetime of ROS within tissues can be overcome effectively, for CR@Alg microrobots carrying photosensitiser, chlorophyll, to increase the range of action of ROS under light conditions^47^.

### 3.4 Enhanced penetration of biofilms

*P. aeruginosa* can produce highly structured heterogeneous biofilms, and the extra polymeric substance matrix prevents drug penetration, binding and repelling charged antibiotics^6^. Therefore, it is important to improve the abilities of microrobots to disrupt the structure and infiltrate into the biofilms. Immobilization of alginate lyase allowed CR@Alg microrobots to degrade alginate in biofilms, and thus disperse the dimensional structure of the matrix. Combined with the flexible swimming behavior and the activity of alginate degradation, CR@Alg microrobots can penetrae into the inner biofilms, mending the susceptibility of bacteria inside of the biofilms. To evaluate the biofilm penetration effect of CR@Alg microrobots, *P. aeruginosa* bacteria was placed in 96-well plates to allow the formation of 48 h-biofilm as a model with the thickness of approximately 60 μm depicted in Supporting Information Fig. S7. Leveraging autofluorescence from the chloroplasts in *C. reinhardtii*, the result of 3D fluorescent images showed that a certain number of CR@Alg microrobots efficiently penetrated through the biofilms (Fig. 3F). The results of quantitative fluorescence analysis on the stacked longitudinal sections of *C. reinhardtii* and CR@Alg density in biofilms were shown in Fig. 3G. Compared to *C. reinhardtii* group, around 1.9-fold CR@Alg microrobots invaded into the biofilms with alginate lyase immobilization. As the presented by laser irradiation, more than 2.3-fold CR@Alg microrobots penetrated into the biofilms. Taken together, these results were consistent with the previous reports that alginate lyase has proved to degrade alginate exopolysaccharide^11^. Furthermore, PDT has been demonstrated to damage the components of ECM^35^, and the generated ROS can also promote the penetration of microrobots into biofilms. CR@Alg microrobots upon laser irradiation herein exhibited excellent ability to disperse the architecture of biofilms and enhance bacterial biofilm penetration effect.

### 3.5 *In vitro* biofilm eradication

**Figure 4:**
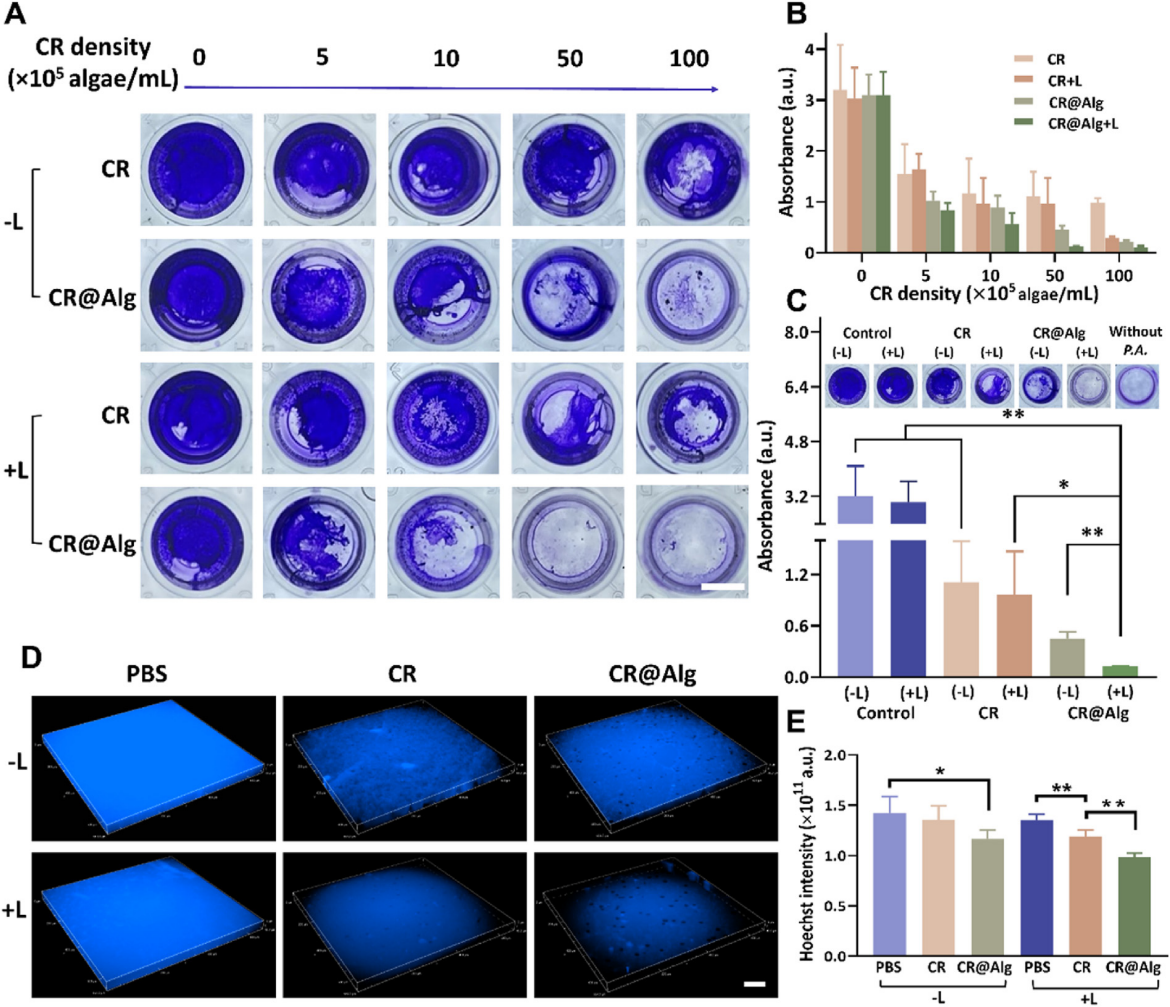
Anti-biofilm effects. (A) Photographs of the *P. aeruginosa* (*P.A.*) biofilms stained by crystal violet, showing the destructive effects of different densities of CR@Alg + Laser treatment on biofilms (scale bar = 5 mm). (B) OD 570 nm value of crystal violet staining for different densities of CR@Alg and (C) each group with the same algae densities (*n* = 4). (D) 3D fluorescence images of *P. aeruginosa* biofilms stained with Hoechst (live cells, blue channel) in the control group, only laser group, *C. reinhardtii* group, *C. reinhardtii* + Laser group, CR@Alg group and CR@Alg + Laser group (scale bar = 100 μm). Laser groups were treated with a 638 nm laser (0.6 W/cm^2^, 30 min). (E) Mean fluorescence intensity of *P. aeruginosa* biofilms in different groups. Data are presented as mean ± SD (*n* = 4). ∗*P* < 0.05, ∗∗*P* < 0.01.

Encouraged by the results of penetration, the biofilm eradication ability of CR@Alg microrobots was further investigated. Compared with the passive particles, microalgae have higher mechanical energy to attack biofilms due to their flagellar oscillations, supporting actively swimming motion. Meanwhile, ROS generated from the chlorophyll of *C. reinhardtii* disrupted biomolecules of bacteria and resulted in bacteria death^49^^,^^50^. Their activity of biofilm eradication was detected by crystal violet and Hoechst staining methods. After different treatments, the mature biofilms were stained by crystal violet, and thus they can be observed intuitively at 570 nm to evaluate the enhanced PDT efficacy of CR@Alg microrobots. As illustrated in Fig. 4A, the biofilm-destructive effect of CR@Alg microrobots were substantially elevated upon 638 nm laser irradiation by comparing the photographs of crystal violet-stained biofilms. Biofilms became loose and porous after treating with CR@Alg and CR@Alg + Laser, and almost no biofilm was observed in wells at density of 5.0 × 10^6^ and 1.0 × 10^7^ algae/mL in CR@Alg + Laser group. In parallel, the stained biofilms of each group and different densities of *C. reinhardtii* were dissolved and then measured at 570 nm. Comparing the OD 570 nm values of each group, the CR@Alg microrobots exhibited the strongest penetrating and anti-bacterial effects under laser irradiation, generating 10.7- and 1.6-fold higher eradication effect than the control group and *C. reinhardtii* group at the density of 5.0 × 10^6^ algae/mL (Fig. 4B and C and Supporting Information Fig. S8).

In a microcosmic perspective, 3D images of Hoechst staining DNA of bacterial cells demonstrated the eradication effect of CR@Alg + Laser treatment on biofilms intuitively (Fig. 4D). Notably, compared with the fluorescent images of other groups, the biofilms became the thinnest and the weakest after treating with CR@Alg + Laser. The results were further confirmed by the quantitative analysis of fluorescence intensity by Image J and showed that CR@Alg + Laser group was 1.4-fold lower than that of the control group (Fig. 4E). Taken together, the treatment of CR@Alg with laser irradiation demonstrates that the combination of ROS and alginate lyase dispersed the architecture of biofilms, and caused much more distinct destructive performance on *P. aeruginosa* bacteria *in vitro*.

### 3.6 *In vivo* anti-bacterial activity and anti-inflammatory

**Figure 5:**
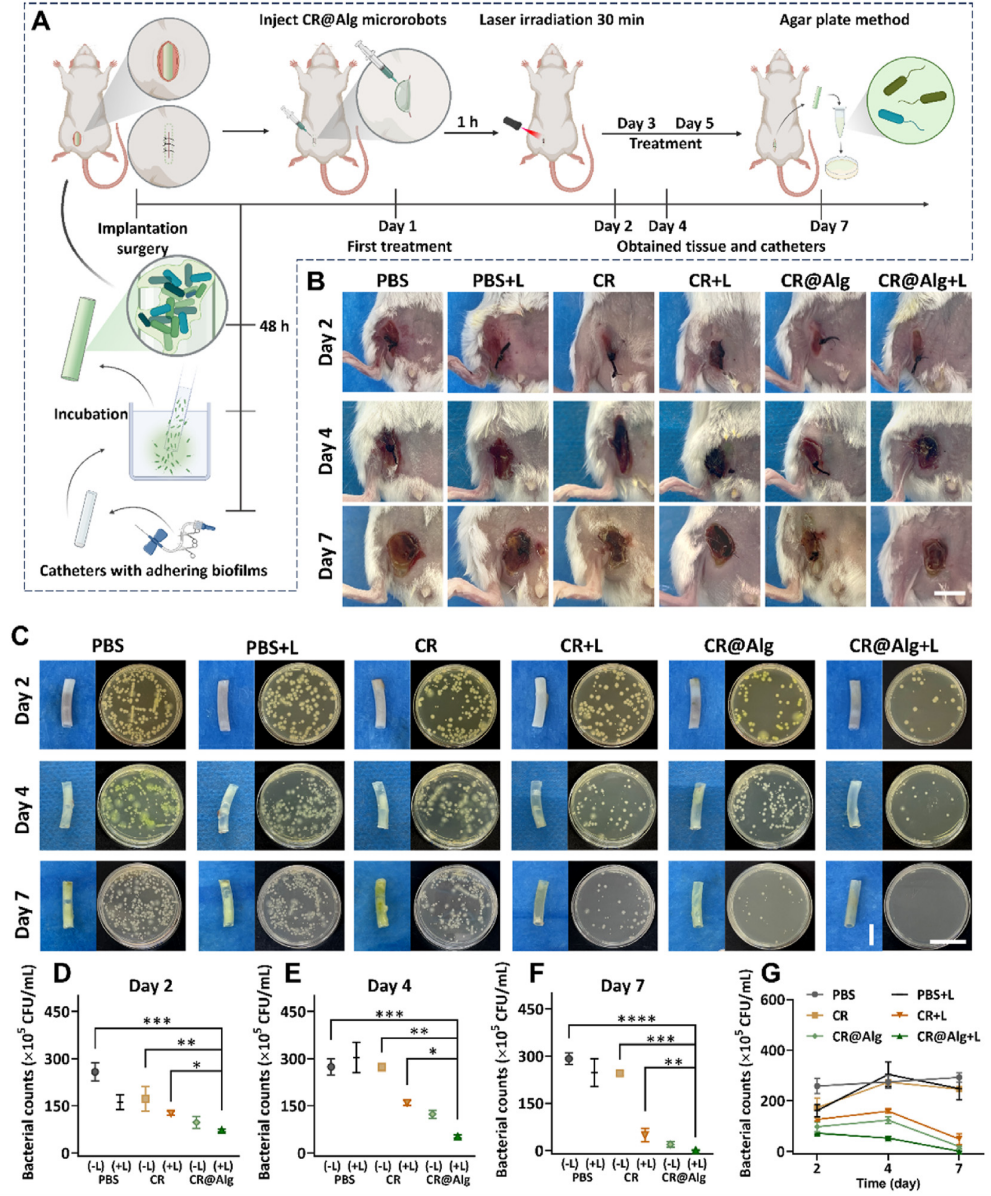
*In vivo* antibacterial effect. (A) Experimental schematic of implantation surgery and CR@Alg microrobots antibacterial treatment in infected mice. (B) Photographs of the incision areas (scale bar = 10 mm), (C) catheters removed from original surgery sites in each group (scale bar = 5 mm), and bacterial colonies (scale bar = 45 mm) from mice under different treatments on Days 2, 4, and 7. (D–F) Quantitative analysis of bacterial colony-forming unit (CFU) obtained from implanted catheters in each group, data are presented as mean ± SD (*n* = 3). (G) Curves of the bacterial CFU after different treating 1, 2, and 3 times. ∗*P* < 0.05, ∗∗*P* < 0.01, ∗∗∗*P* < 0.001 and ∗∗∗∗*P* < 0.0001.

Before investigating the *in vivo* anti-bacterial effect, safety issues have also been considered. *C. reinhardtii* show good biocompatibility^25^ and their abundant chlorophyll can generate ROS under specific laser irradiation^31^ rather than photosensitizer of inorganic–organic hybrid materials with potential toxicity^51^. Based on the *in vitro* data, CR@Alg microrobots had been already validated the effect of ECM degradation by immobilized alginate lyase and the excellent locomotion, as an active natural photosensitizer delivering platform to enhance PDT at the depth of biofilms. We further demonstrated the short-term bacterial eradicating and anti-inflammatory effects of CR@Alg microrobots synergistic therapy on an implanted catheter infective mouse model (Fig. 5A). The body weights of BALB/c mice were recorded before implanting catheters with adhering biofilms on Day 0 and after the implantation. As shown in Supporting Information Fig. S9A and S9B, the body weights of the mice were decreased by 10% on Day 1 before treatment, redness and swell occurred at the surgical site of skin, which showed that the model of acute infection was established. Subsequently, the infected mice were randomly divided into 6 groups (*n* = 4) including PBS, PBS + Laser, *C. reinhardtii*, *C. reinhardtii* + Laser, CR@Alg, CR@Alg + Laser groups, and the mice were respectively administrated on Days 1, 3 and 5 (Fig. 5A). Compared with the infected sites on Day 0 (Supporting Information Fig. S10), the infected sites became rubor or swollen in all of groups before treatment on Day 1. As time passed, better healing of infected wounds was observed after treating with CR@Alg + Laser on Day 7, whereas the ulcer occurred in infected sites and became increasingly inflamed in PBS group (Fig. 5B). During the treatment, the body weights of the mice were monitored and the weights recovered to normal level more effectively under the treatment of CR@Alg microrobots, indicating no acute toxicity of CR@Alg microrobots (Supporting Information Fig. S9A and S9B).

Moreover, to clearly identify the effects of bacteria elimination by CR@Alg microrobots, the bacteria were obtained from implanted catheters, and evaluated quantitatively by using the agar plate dilution method. As shown in Fig. 5C, gradually serious pyosis in the implanted catheters of PBS group and PBS + Laser group were observed on Days 4 and 7, indicating that the infection and inflammations were mainly caused by bacteria without CR@Alg + Laser treatment. By contrast, the implanted catheters of CR@Alg + Laser group were obviously cleaner, and the pyosis almost disappeared on Day 7. On the other hand, the agar plate dilution method was employed to measure the colony forming unit (CFU) of implanted catheters and verify the biofilm eradication effect of CR@Alg microrobots quantitatively. As shown in Fig. 5D‒G, the number of live bacteria in the treated biofilms was considerably reduced, and bacteria were almost not detected on the surface of the catheters under the treatment of CR@Alg + Laser on Day 7, whereas the agar plate data of PBS group saw a gradual rise between Days 2 and 7. Besides, although the PBS + Laser group showed decreased bacterial counts on Day 2 and probably depended on the promoted microbicidal activity of phagocytes, triggered by photobiomodulation (PBM)^52^, this only laser therapy way failed to inhibit bacterial activity and hard to eradicate the bacterial biofilms for the rest of the time of this infective model. Furthermore, biofilms were degraded by CR@Alg, and the degradation products of alginate, reported as alginate oligosaccharides (AOs), have been found that they exhibit immunomodulatory physiological activities^14^. Also, once the bacteria lose the protection of biofilms, they are faced with the secondary metabolites^53^ generated by microalgae and phagocytes clearing behavior *in vivo* environment. Therefore, as shown in Fig. 5C and F, it was a combination approach of CR@Alg *in vivo* treatment to eliminate bacteria and becomes the basic function for enhancing anti-bacterial laser therapy. But compared with CR@Alg + Laser group, CR@Alg still needs laser irradiation to achieve a better bacterial-eliminating effect for the whole period of treatment. Accordingly, CR@Alg with laser therapy can enhance the anti-bacterial effect remarkably for the whole 7-day period of the bacterial biofilm-infective model, indicating that CR@Alg microrobots enhanced photodynamic effect and performed a biofilm elimination potency *in vivo*.

**Figure 6:**
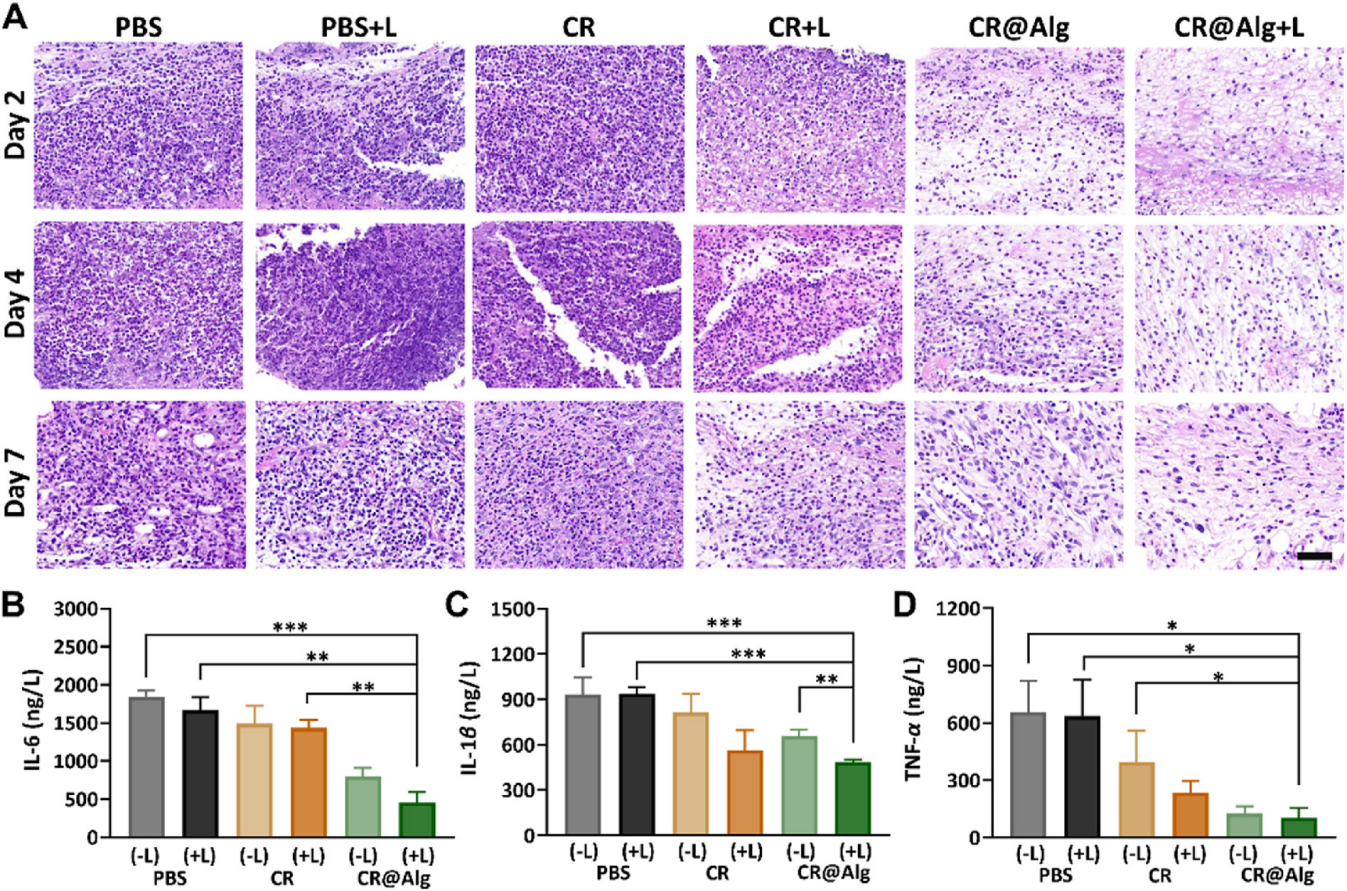
*In vivo* anti-inflammatory effects. (A) H&E staining of the tissues around the implanted sites on Days 2, 4, and 7. Neutrophils tended to be produced in the infection sites and were stained blue (scale bar = 50 μm). (B–D) Quantitative analysis of the level of IL-6, IL-1*β,* and TNF-*α* in the tissues on Day 4 after different treatments. Data are presented as mean ± SD (*n* = 3). ∗*P* < 0.05, ∗∗*P* < 0.01, ∗∗∗*P* < 0.001.

Since the lipopolysaccharide (LPS) in the gram-negative bacterium elicits inflammatory cell infiltration and up-regulates inflammatory cytokines level^54^, the intrinsic immune response of the implanted catheter mouse model was mainly triggered by bacterial infection. Furthermore, to investigate the antibacterial effect and anti-inflammatory effect, the tissues around the infected regions of the mice were harvested and stained with hematoxylin and eosin (H&E). It was found that neutrophils tended to aggregate in the infection sites. As depicted in Fig. 6A, fewer neutrophils were observed in the CR@Alg + Laser group, indicating that the inflammatory reaction was milder. Conversely, severe neutrophilic infiltration was observed in the PBS group and PBS + Laser group, which demonstrated obvious acute inflammation because of CR@Alg + Laser treatment absence after bacterial infection. We also quantitatively assessed the changes in inflammatory cytokines on Day 4 after different groups’ treatment two times as a surrogate of recovery in mice. As displayed in Fig. 6B‒D, the inflammatory cytokines levels of IL-1*β*, TNF-*α*, and IL-6 in the CR@Alg (dark) and CR@Alg + Laser groups were greatly reduced, compared to that of those in the PBS group. In contrast to bacterial flagellin^55^, further *in vivo* studies verified that CR@Alg microrobots prevent innate immune cells producing proinflammatory cytokines. As previous studies reported that microalgae can use a range of strategies to withstand stress, including phenolic compounds belonging to secondary metabolites^53^, which possess anti-inflammatory activity^56^. The secondary metabolites of *C. reinhardtii* can possibly inhibit inflammation and clean away the inflammatory chemokines. Therefore, this microalgae-based therapy would offer prospective benefits for the treatment of important diseases. These results also indicated that CR@Alg microrobots did not trigger the significant production of proinflammatory cytokines, consistent with the previous report^30^. Simultaneously, CR@Alg microrobots performed prominent antibacterial and anti-inflammatory effects *in vivo*, exhibiting great prospects for clinical application.

Moreover, the main organs of the mice in both the experimental and control groups were fixed, using H&E staining to evaluate the potential toxicity (*n* = 4). The results indicated that the administration of the CR@Alg microrobots did not induce any pathogenic pathological effect on the separated tissues from the BALB/c mice at the tested concentration when compared to the PBS group (Supporting Information Fig. S11). Also, CR@Alg microrobots did not result in significant harm to the mice, indicating the feasibility of their utilization in the treatment of *in vivo* bacterial infections.

## 4 Conclusions

In summary, we demonstrated biohybrid microalgae-based robots with a biofilm eradication ability and biocompatibility by immobilizing alginate lyase *via* the biological orthogonal method. This method preserved both microalgae and alginate lyase bioactivities, and thus the CR@Alg microrobots can be readily fabricated by a biotin−streptavidin−biotin approach, without compromising the motion behavior of algae and the bioactivity of alginate lyase. After the modification on the surface of *C. reinhardtii* with alginate lyase, CR@Alg microrobots were allowed to penetrate the bacterial biofilms much easier, due to the enhanced capability to degrade alginate of biofilm matrix. When the self-propelled CR@Alg microrobots swam into the depth of the biofilms, the alginate lyase also reached because of the bioconjugation with *C. reinhardtii* and continued to degrade the biofilms. Moreover, thanks to the abundant chlorophyll in *C. reinhardtii*, as a natural photosensitizer, CR@Alg microrobots generated a large amount of ROS under a 638 nm laser irradiation, to achieve synergistic bactericidal as well as anti-inflammatory effects *in vivo* in the implanted catheter mouse model associated with biofilm formation on urinary and central venous catheters. Therefore, CR@Alg microrobots offered an approach to healthcare for catheter-implanted patients who can just accept minimally invasive surgical or medical treatments. Our work developed minimally invasive CR@Alg microrobots with promising biofilm eradication ability, providing a potential strategy without antibiotics and surgical debridement to eliminate medical device-associated biofilm infections.

## Author contributions

Xiaoting Zhang: Conceptualization, Formal analysis, Data curation,Writing – original draft, Methodology, Investigation. Huaan Li: Conceptualization, Supervision, Formal analysis, Methodology, Investigation. Lu Liu: Methodology, Investigation. Yanzhen Song: Methodology, Investigation. Lishan Zhang: Methodology, Formal analysis. Jiajun Miao: Formal analysis. Jiamiao Jiang: Formal analysis. Hao Tian: Formal analysis. Chang Liu: Supervision. Fei Peng: Supervision. Yingfeng Tu: Writing – review & editing, Supervision, Funding acquisition, Conceptualization.

## Conflicts of interest

The authors declare no conflict of interest.

## Appendix ASupporting information

The following is the Supporting Information to this article:
