# Supplementary material for: Alginate lyase immobilized Chlamydomonas algae microrobots: minimally invasive therapy for biofilm penetration and eradication
Source: Acta Pharm Sin B. 2025 Mar 18;15(6):3259–72. doi: 10.1016/j.apsb.2025.03.034 (PMC12254743; doi:10.1016/j.apsb.2025.03.034)
Supplement: Multimedia component 1 [file mmc1.docx]

**Supporting Information for**

**ORIGINAL ARTUCLE**

**Alginate lyase immobilized *Chlamydomonas* algae microrobots: minimally invasive therapy for biofilm penetration and eradication**

**Xiaoting Zhang ^a, †^, Huaan Li ^b, †,^ *, Lu Liu ^a^, Yanzhen Song ^a^, Lishan Zhang ^a^, Jiajun Miao ^a^, Jiamiao Jiang ^a^, Hao Tian ^a^, Chang Liu ^c,^ *, Fei Peng ^d,^ *, Yingfeng Tu ^a,^ ***

^a^ *NMPA Key Laboratory for Research and Evaluation of Drug Metabolism & Guangdong Provincial Key Laboratory of New Drug Screening, School of Pharmaceutical Sciences, Southern Medical University, Guangzhou 510515, China.*

^b^ *Guangdong Provincial Key Laboratory for Research and Evaluation of Pharmaceutical Preparations & Guangdong Provincial Engineering Center of Topical Precise Drug Delivery System, Center for Drug Research and Development, Guangdong Pharmaceutical University, Guangzhou 510006, China.*

^c^ *Sport Science College, Beijing Sport University, Beijing 100091, China*

^d^ *School of Materials Science and Engineering, Sun Yat-Sen University, Guangzhou 510275, China.*

*Corresponding Author

Tel./fax: +86 18898631797 (Yingfeng Tu)

E-mail: tuyingfeng1@smu.edu.cn (Yingfeng Tu), pengf26@mail.sysu.edu.cn (Fei Peng), lihuaan@gdpu.edu.cn (Huaan Li), changliu.epigenetic@gmail.com (Chang Liu)

†These authors made equal contributions to this work.

1. **Supplementary figures**


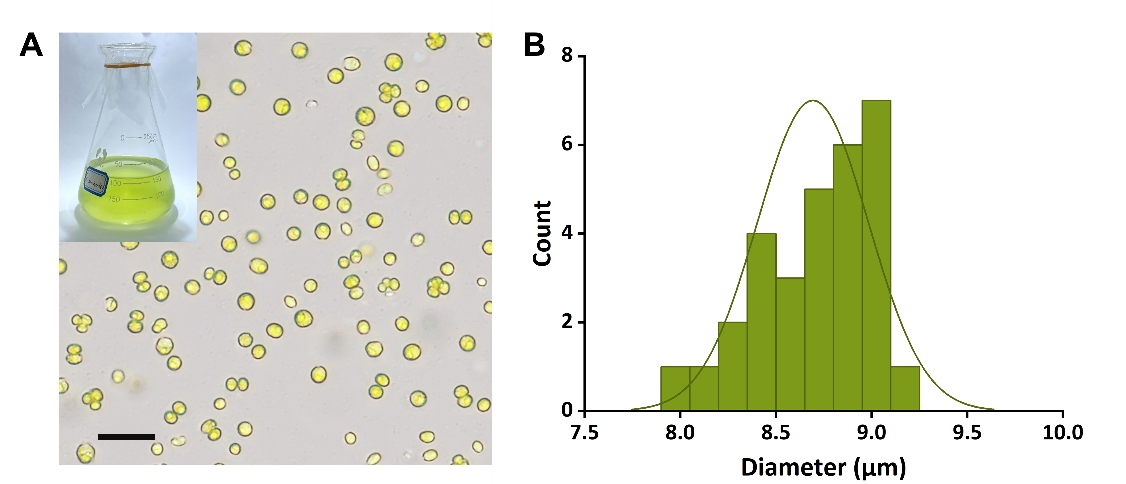


**Figure S1** (A) Photograph of *C. reinhardtii* (inset, large-scale preparation of *C. reinhardtii*) (scale bar= 20 µm) and (B) average diameter of *C. reinhardtii* (*n* = 30).


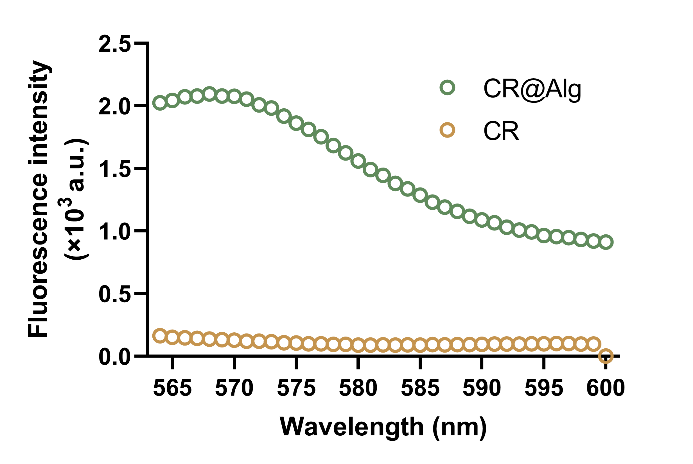


**Figure S2** Fluorescence spectra of *C. reinhardti* and CR@Alg microrobots.


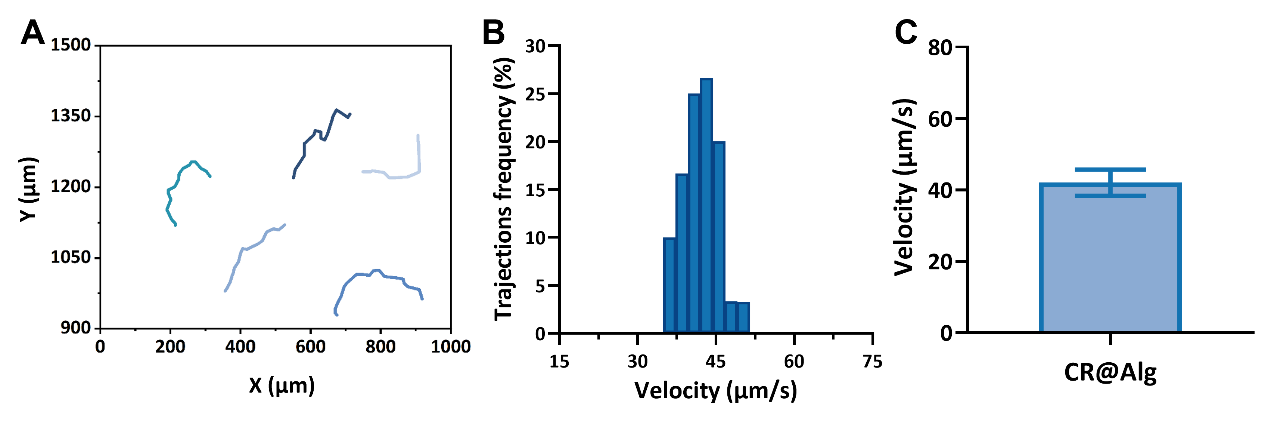


**Figure S3** The motion analyzation of CR@Alg microrobots after 30 min 638 nm laser irradiation with 0.6 W/cm^2^ in PBS at 37 °C. (A) Representative tracking trajectories of CR@Alg microrobots. (B) Mean velocity distribution of CR@Alg microrobots. (C) The velocity of CR@Alg microrobots (*n* = 100).


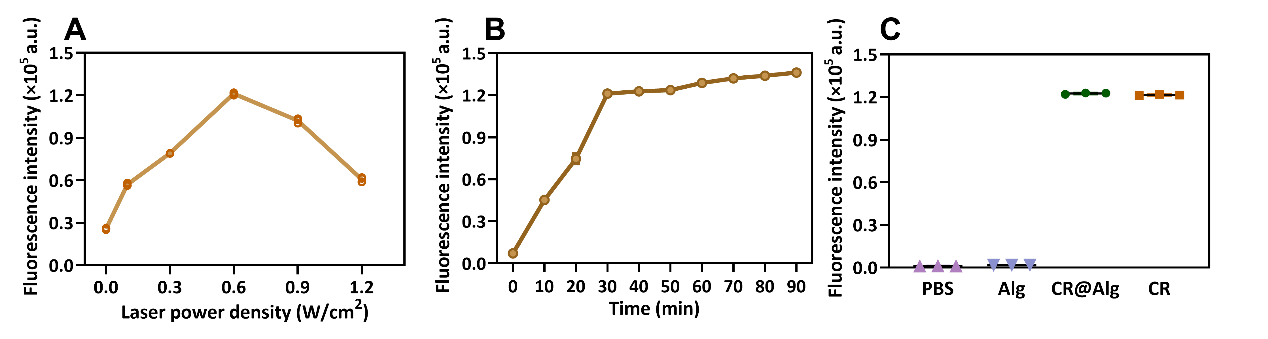


**Figure S4** (A) Fluorescence intensity of DCF in *C. reinhardtii* (5.0×10^6^ algae/mL) treated by 638 nm laser irradiation with different power density for 30 min. (B) Fluorescence curves of DCF in *C. reinhardtii* (5.0×10^6^ algae/mL) irradiated by 638 nm laser with 0.6 W/cm^2^. (C) Fluorescence intensity of DCF in CR and CR@Alg (5.0×10^6^ algae/mL), PBS medium, treated by 638 nm laser irradiation with 0.6 W/cm^2^ for 30 min (*n* = 3). Data are presented as mean ± SD.


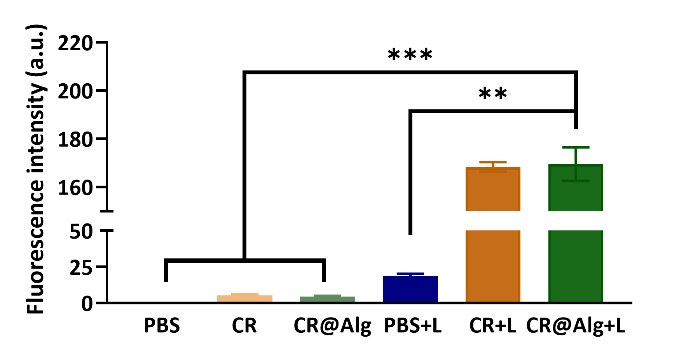


**Figure S5** Quantitative analyzed to assess DCF fluorescence intensity of *P. aeruginosa* cells in different groups. Data are presented as mean ± SD (*n* = 4). **P* < 0.05, ***P* < 0.01, ****P* < 0.001.


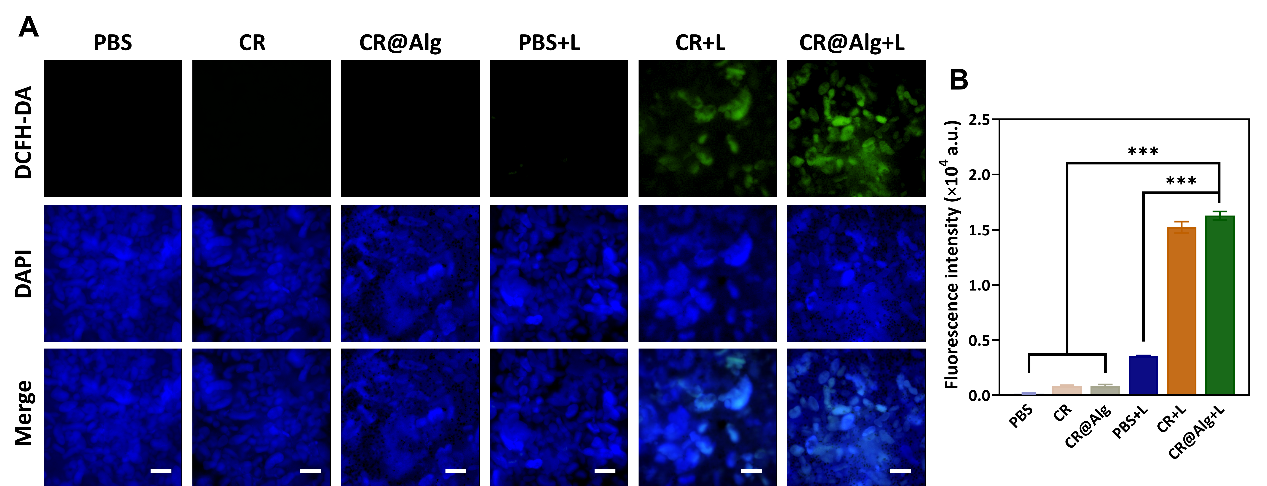


**Figure S6** (A) DCFH-DA and DAPI staining fluorescence images of ROS in *P. aeruginosa* biofilms in different groups (scale bar = 100 μm). (B) Quantitative analyzed to assess DCF fluorescence intensity of *P. aeruginosa* biofilms in different groups. Data are presented as mean ± SD (*n* = 4). **P* < 0.05, ***P* < 0.01, ****P* < 0.001.


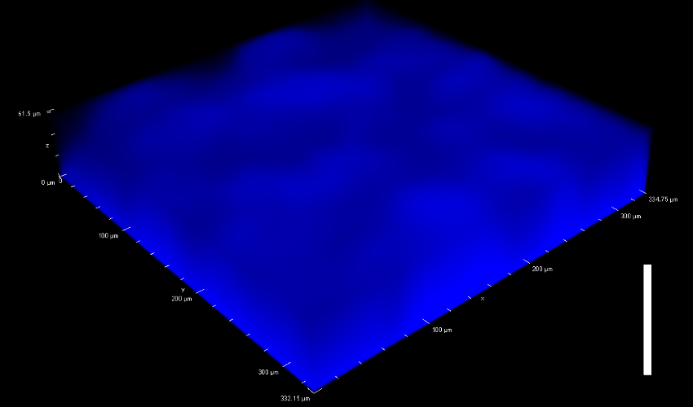


**Figure S7** 3D fluorescence image of 48 h-cultivated *P. aeruginosa* biofilms (DAPI staining, blue) (scale bar = 100 μm).


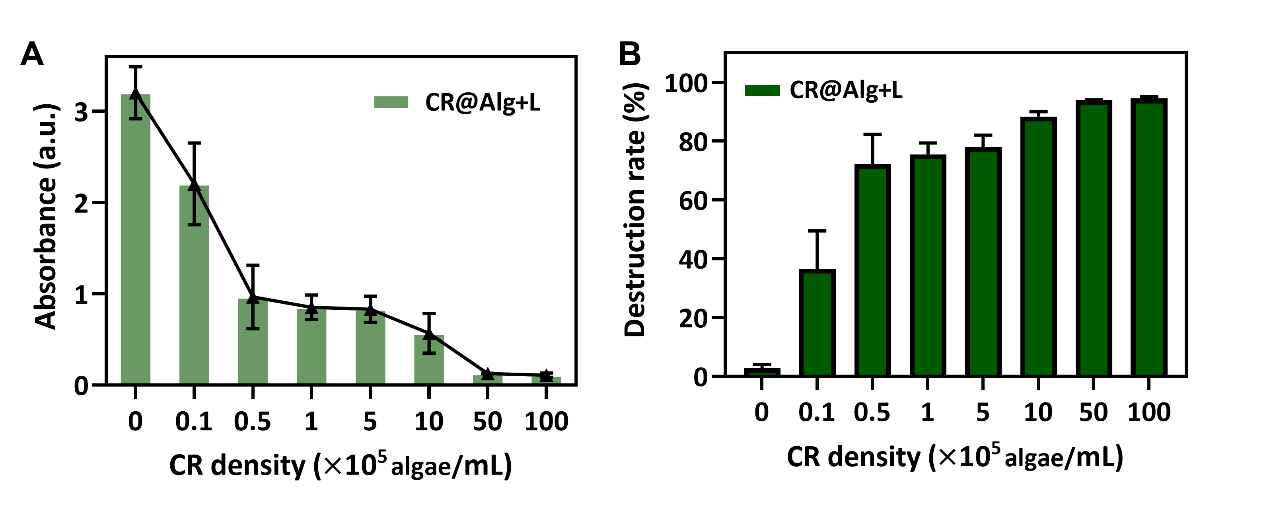


**Figure S8** Destructive effects of CR@Alg + Laser treatment on biofilms. (A) OD 570 nm value of crystal violet staining for biofilms. (B) Destruction rate of different densities of CR@Alg microrobots. Data are presented as mean ± SD (*n* = 4).


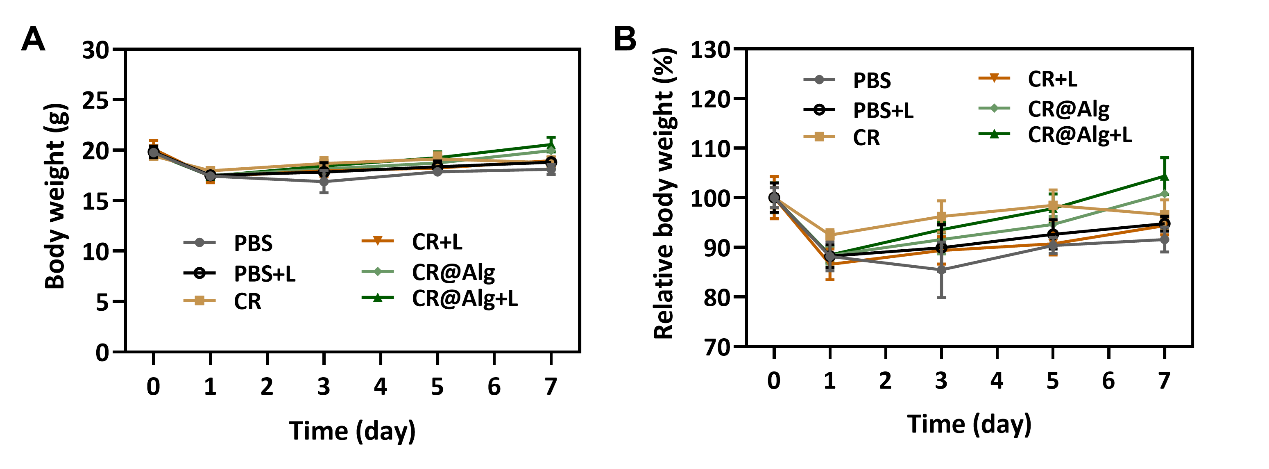


**Figure S9** (A) Body weights and (B) the relative body weights of BALB/c mice after different treatments. Data are presented as mean ± SD (*n* = 4).


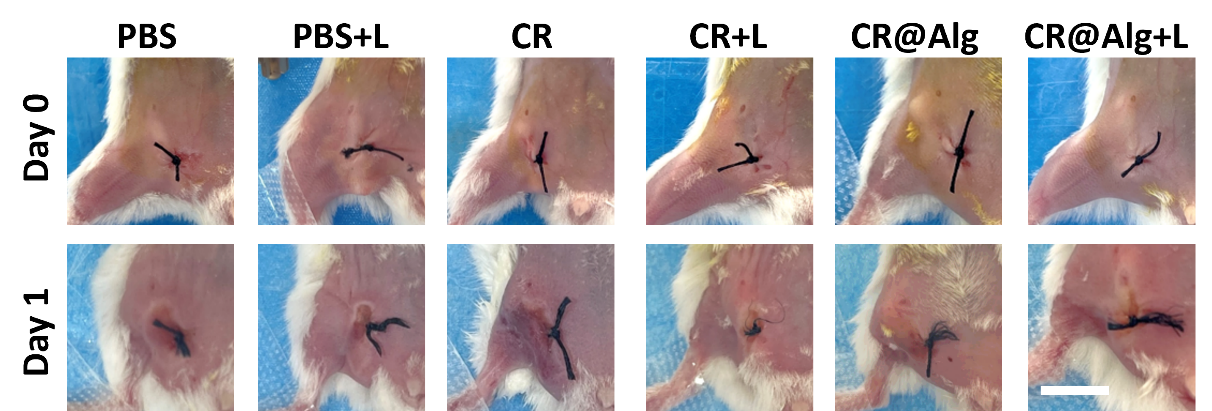


**Figure S10** Photographs of the incision areas of mice after implantation surgery (scale bar = 10 mm).


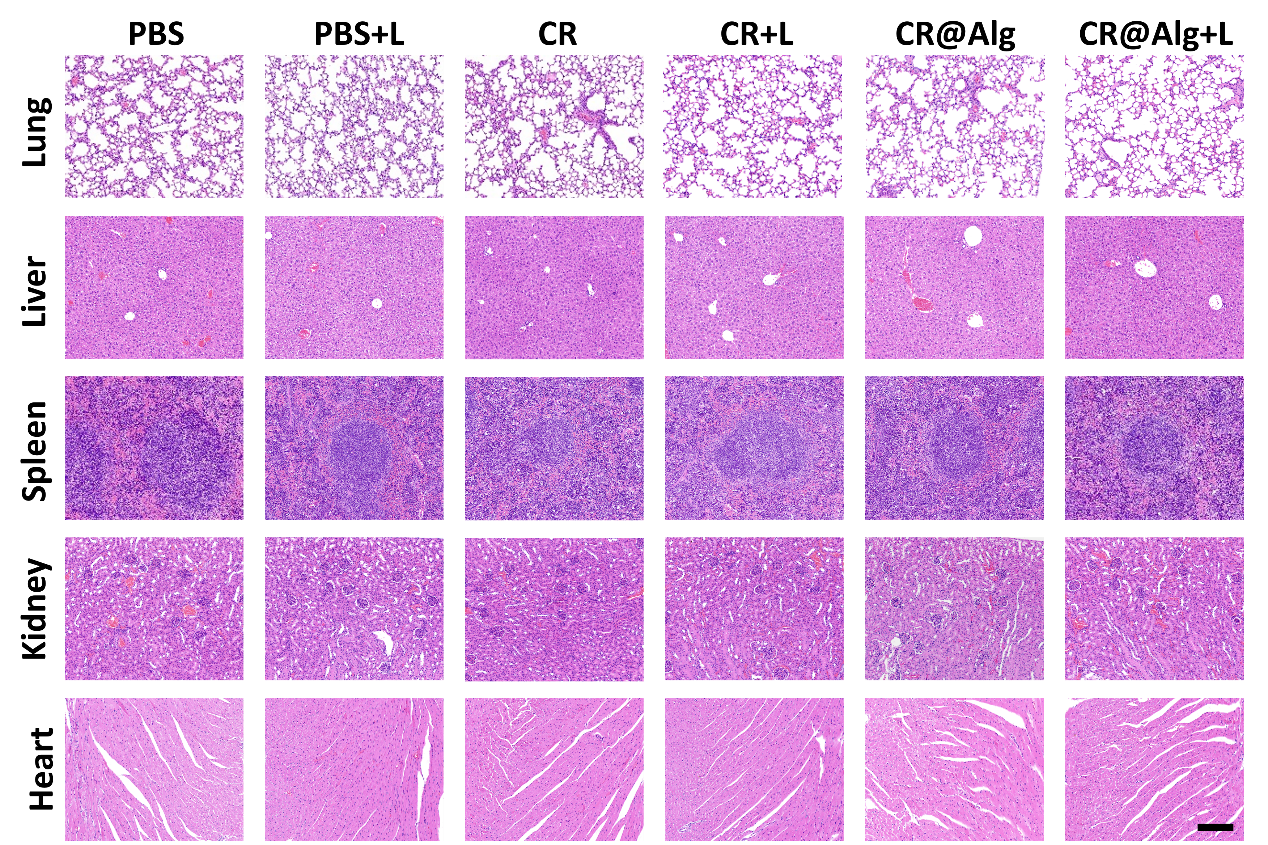


**Figure S11** H&E staining of lung, liver, spleen, kidney and heart tissue slices from different groups after treatment (scale bar = 50 μm).
